# Outcome of antithrombotic therapy in cancer patients with catheter-related thrombosis: a systematic review

**DOI:** 10.3389/fcvm.2023.1290822

**Published:** 2023-12-12

**Authors:** Qinan Yin, Xingyue Zheng, Lizhu Han, Xuefei Huang, Yueyuan Wang, Yujie Song, Yuan Zhang, Yuan Bian

**Affiliations:** ^1^ Department of Pharmacy, Sichuan Academy of Medical Sciences & Sichuan Provincial People’s Hospital, Chengdu, China; ^2^ Personalized Drug Therapy Key Laboratory of Sichuan Province, School of Medicine, University of Electronic Science and Technology of China, Chengdu, China

**Keywords:** catheter-related thrombosis, anticoagulation, cancer patients, systematic review, VKAs, DOACs, LMWH

## Abstract

**Introduction:**

The guidelines' recommendations for anticoagulation in cancer patients with catheter-related thrombosis are unclear. The aim of this systematic review was to assess anticoagulation management in cancer patients with catheter-related thrombosis (CRT) based on previously published studies.

**Methods:**

As of June 10, 2023,we searched databases including PubMed, Embase, and Cochrane and included 11 observational studies that met the criteria. We evaluated 770 adults with active cancer and objectively confirmed patients with CRT who were using drugs including warfarin, LMWH, and new oral anticoagulants as antithrombotic therapy.

**Results:**

We extracted outcome data, including thrombosis recurrence, catheter dysfunction, major bleeding, and death, and performed a meta-analysis.

**Discussion:**

In this study we found that the risk of VTE recurrence was higher with rivaroxaban, the risk of bleeding and death appeared to be greater with warfarin, and although the risk of catheter dysfunction due to LMWH is a concern, it is still a more reasonable option for cancer patients with catheter-related thrombosis.

**Systematic Review Registration:**

http://www.clinicaltrials.gov, identifier (CRD42022367979).

## 1. Introduction

Central venous access devices (CVADs) are a current treatment option for patients with cancer and critical illness. The main types of CVADs currently used in patients include central venous catheters (CVCs) and peripherally inserted central catheters (PICCs). Catheter-related thrombosis (CRT) is a special type of venous thromboembolism (VTE), and the common symptoms are limb pain and edema. CVAD-associated intimal damage activates platelets and the coagulation system, leading to local vasoconstriction, platelet adhesion, and fibrin generation, which can lead to thrombosis (1). In addition, persistent endothelial injury and thrombosis are further facilitated by continuous friction between CVAD and the vessel wall, turbulence in the catheter, and toxic effects of certain drugs (2). Finally, CRT can lead to recurrent deep venous thrombosis (DVT), postthrombotic syndrome (PTS), pulmonary embolism (PE), and sepsis (3). There is no hard data on the incidence risk of venous thrombosis associated with CVC or PICC, but the rate of PICC-associated CRT appears to be a lower (4). Cancer is an important risk factor for developing venous thromboembolism. The prevalence of asymptomatic CRT in cancer patients varied among studies, with the meta-analysis reporting a prevalence of 6.67% (5, 6). Compared with noncancer patients, there is a delicate balance between thrombosis and bleeding in cancer patients, with a 3-fold higher risk of thrombosis recurrence and a 2-fold higher risk of major bleeding (7).

The risk factors associated with CRT include not only the type of catheter but also the diameter of the catheter and the number of lumens. Larger-diameter central venous catheters are more likely to cause thrombosis than smaller-diameter catheters. For a variety of PICC catheters of different diameters, it was found that the probability of DVT in 5Fr PICC catheters was significantly higher than that in 4Fr PICC catheters (8). The position and side of the catheter also affect the risk of thrombosis. The ONCOCIP study is a French prospective multicenter study that analyzed risk factors for thrombosis implantation in patients with solid tumors. The results of the multivariate analysis showed that the use of a cephalic vein insertion catheter was associated with an increased risk of CRT (9). Compared with right CVCs, left catheter insertion has an increased risk of thrombosis (10, 11). If the patient has no anticoagulation contraindications, anticoagulation therapy is recommended regardless of whether the catheter is removed (3). To alleviate patient symptoms and reduce the risk of recurrent thrombosis and pulmonary embolism, the European Society for Vascular Surgery (ESVS) guidelines recommend anticoagulation with low molecular weight heparin (LMWH) or LMWH in combination with vitamin K antagonists (VKA) for at least 3 months in patients with CRT (3). Based on the availability of direct oral anticoagulants (DOACs) in CRT patients, we completed a systematic review to assess the impact of DOACs on thrombotic recurrence, catheter dysfunction, bleeding risk, and death compared to LMWH and warfarin.

## 2. Materials and methods

### 2.1. Search strategy and study selection

Databases including PubMed, Embase and Cochrane were searched through June 10, 2023. Clinical Trials (http://www. clinicaltrials.gov) were also searched. The search criteria included “catheter-related thrombosis”, “cancer patient”, “anticoagulant therapy”, “new oral anticoagulants”, “direct oral anticoagulants”, “rivaroxaban”, and “edoxaban”. “apixaban”, “dabigatran”, “low molecular weight heparin”, “LMWH”, “VKAs”, “warfarin”, “prospective studies”, “retrospective studies”, “CRT”. Titles and abstracts of all retrieved citations were screened by two independent reviewers (Y.Q. N and Z.X. Y) to identify all potentially eligible studies. We retrieved the full text of the relevant content, and any resulting discrepancies could be resolved by discussion involving a third reviewer if necessary. This study was registered in PROSPERO (CRD42022367979).

### 2.2. Inclusion and exclusion criteria

#### 2.2.1. Inclusion criteria

(1) Adults (18 years of age or older) were diagnosed with active malignancy or had metastatic disease and were actively treated. (2) Patients used catheters as part of their cancer management. (3) Confirmation of thrombosis by objectively confirmed symptomatic CRT, such as Doppler ultrasonography, venography, or computed tomography (CT). (4) Therapeutic anticoagulants, including warfarin, low molecular heparin, or new oral anticoagulants, were used as treatments for thrombosis.

#### 2.2.2. Exclusion criteria

(1) The catheter is a dialysis catheter, and the risk of active bleeding or major bleeding is high. (2) CVC had been removed before anticoagulation was initiated or the patient had been using another anticoagulant for more than 7 days before catheter use. (3) Need for dual antiplatelet therapy (e.g., recent coronary stents).

### 2.3. Data extraction and risk of bias assessment

Two reviewers (Y.Q. N and Z.X. Y) independently screened titles and abstracts and reviewed the full text. Articles that met the criteria were included by reviewing the full text. Then, we extracted study characteristics, baseline characteristics, and predetermined efficacy and safety results. The selected studies were scored according to the Newcastle‒Ottawa Quality Assessment Scale (NOS) with a full score of 9, including 4 points for object selection, 2 points for comparability, and 3 points for investigation and assessment methods of exposure. A score ≥5 was considered acceptable for human analysis, and a score ≥7 was considered high-quality research.

### 2.4. Statistical analysis

Stata 17.0 software was used for data analysis, and heterogeneity was assessed using the I^2^ statistic. The pooled effect size and 95% confidence intervals of all results were calculated according to the sample size and number of events studied. If there was no heterogeneity between outcomes (*I*^2^ ≤ 50%, *P *≥ 0.1), we extracted and combined data from cohort studies and performed meta-analyses using a fixed-effects model. If there was heterogeneity among the results (*I*^2^ > 50%, *P *< 0.1), the source of heterogeneity was analyzed first, and sensitivity analysis was conducted, i.e., deleting one study at a time to estimate whether the results would have been significantly affected by a single study, then the random effect model was used for meta-analysis. Descriptive analysis was used for meta-analysis of data that could not be merged. Publication bias was assessed using Egger's test and Begg's test with Stata 17.0 software. Funnel plots were used to indicate the publication bias (Supplementary Figures S1–S4).

### 2.5. Subgroup analysis

In studies treated with warfarin and rivaroxaban, the duration of follow-up or adverse events was less than three months, while the studies with LMWH had a large difference in follow-up time. To explore the robustness of our results, we used the first three months of extractable outcome events from the studies treated with LMWH for subgroup analysis.

## 3. Results

### 3.1. Study selection and characteristics

**Figure 1:**
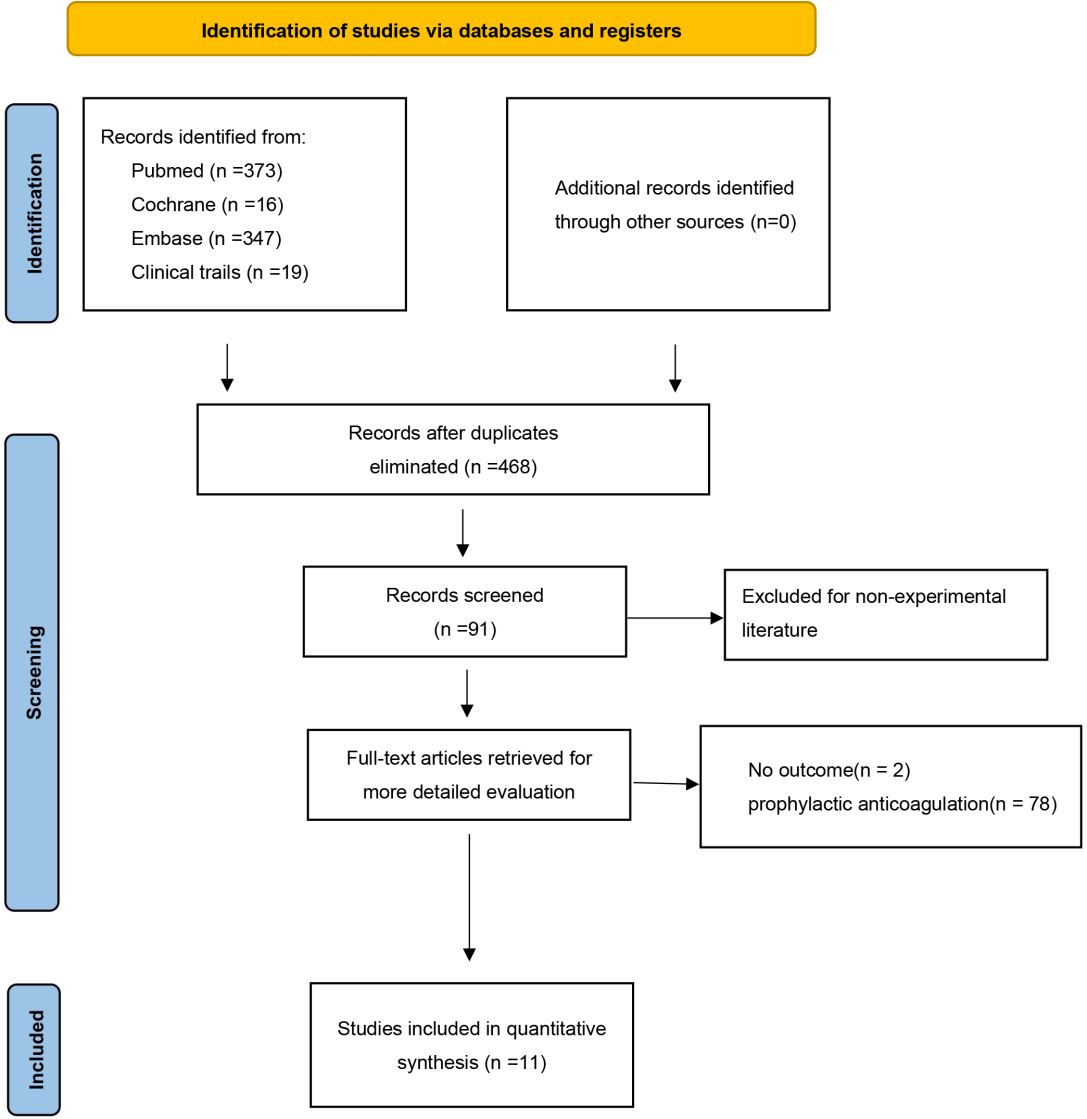
Flow chart of the selected studies.

After screening, 755 potentially eligible studies were identified and were considered for detailed analysis (Figure 1). Eleven studies were eventually included in the meta-analysis: nine were observational cohort studies, and the remaining two were retrospective cohort studies and prospective cohort studies. The characteristics of the included studies are shown in Table 1.

**Table 1:** Clinical characteristics of studies included in the meta-analysis.

Study, year	Study type	Participants	Male (%)	Mean age (years)	Catheter type	Anticoagulation treatment	Time follow up
Kovacs MJ et al. 2007 (12)	Prospective cohort study	74	65	58	PICC (*n* = 59)	Dalteparin + warfari*n*	3 months
Htun KT et al. 2018 (13)	Observation cohort study	23	57	59	PICC (*n* = 10)	LMWH	3 months
Laube ES et al. 2017 (14)	Observation cohort study	83	39	62	PICC (*n* = 5)	Rivaroxaban	90 days
Scamuffa MC et al. 2020 (15)	Observation cohort study	50	58	53.3	PICC (*n* = 50)	LMWH or Fondaparinux	until thrombosis resolution
Davies GA et al. 2018 (16)	Observation cohort study	70	33	54.1	PICC (*n* = 54)	Rivaroxaban	12 weeks
Fan F et al. 2017 (17)	Observation cohort study	84	56%	51.1	PICC	Rivaroxaban (*n* = 41) Enoxaparin + Warfarin(*n* = 35)	3 months
Delluc A et al. 2014 (18)	Observation cohort study	99	68.7	57.3	PICC (*n* = 79)	LMWH (*n* = 94) VKA (*n* = 4) Rivaroxaban (*n* = 2)	632 days (range 6 to 2,495)
Oliver N et al. 2015 (19)	Observation cohort study	35	43	55	PICC (*n* = 33) CVC(*n* = 2)	Enoxaparin	6 months (range, 0–25 months)
Baumann KL et al. 2022 (20)	Observation cohort study	27	59	59	/	Enoxaparin	6 months
Kang J et al. 2016 (21)	Observation cohort study	8	0	48	PICC	LMWH	until PICC was removed
Xu J et al. 2023 (22)	Retrospective cohort study	217	42.4	56	PICC	LMWH(*n* = 118) Rivaroxaban(*n* = 99)	6 months

/: Not specified.

### 3.2. Risk of bias

**Figure 2:**
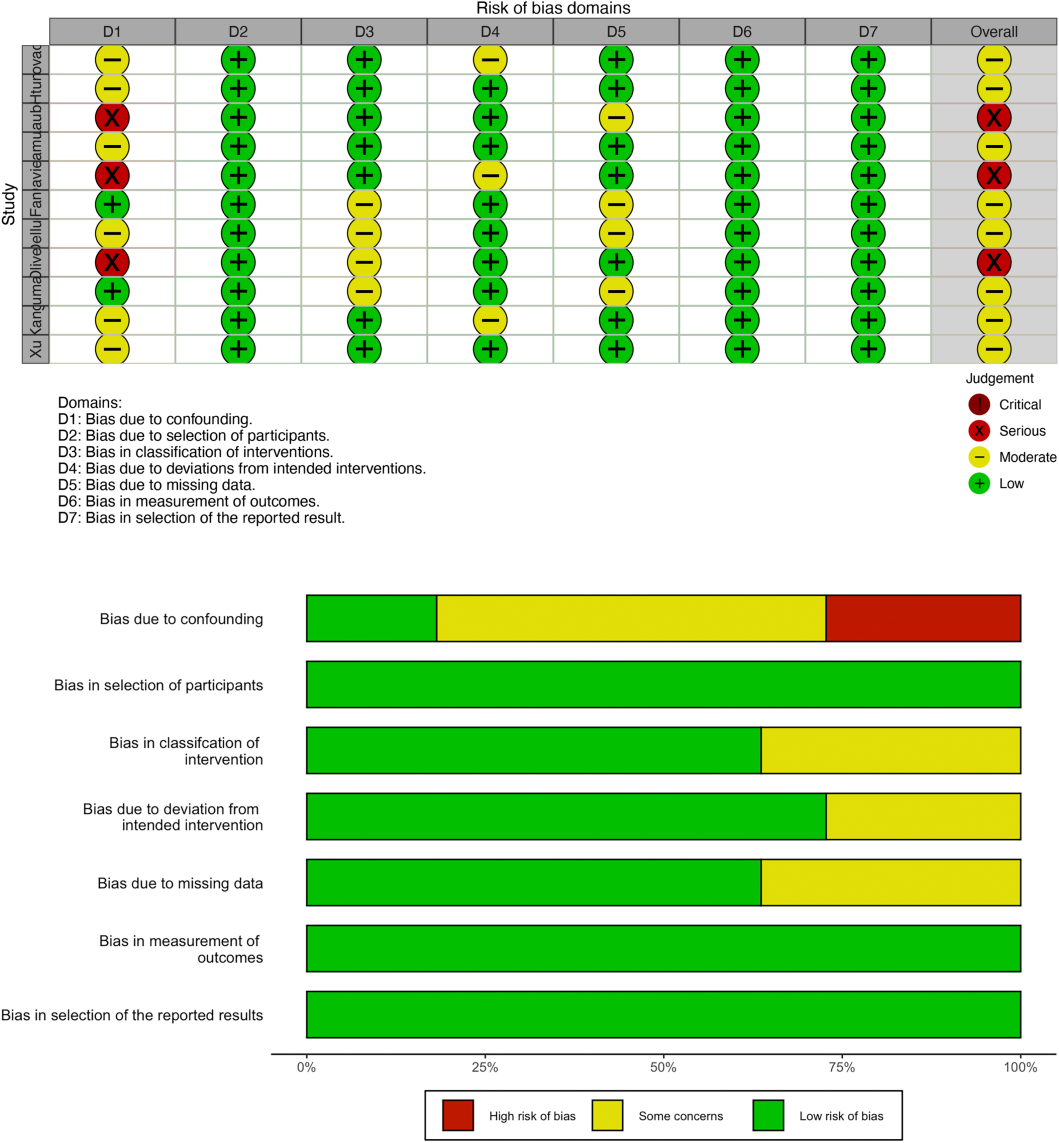
The risk of bias in the included trials.

All included studies were cohort studies with a low risk of bias, which was assessed by the R program (Figure 2). No publication bias was detected in this study.

### 3.3. VTE recurrence

#### 3.3.1. Warfarin

**Figure 3:**
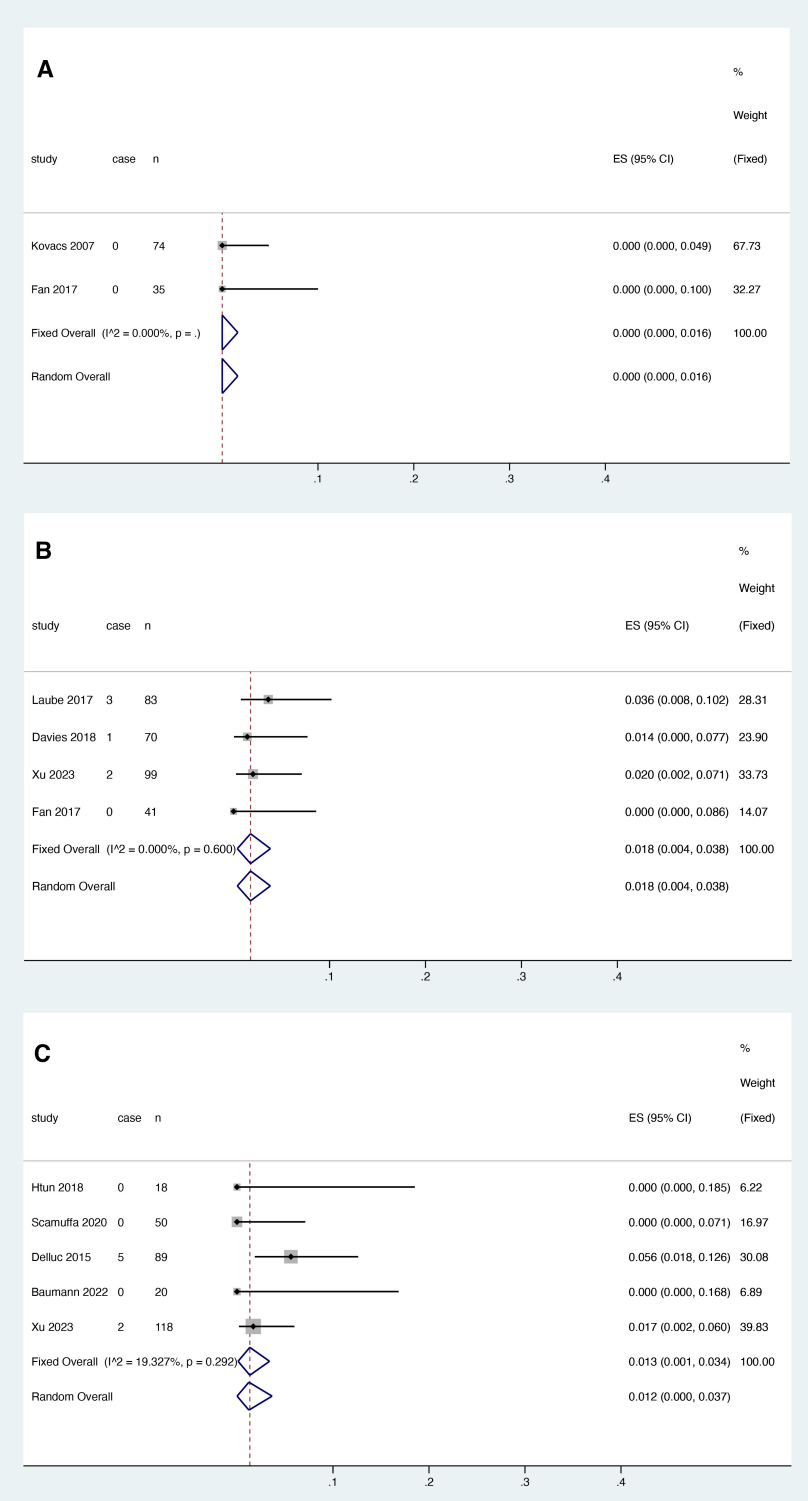
VTE recurrence risk in patients after anticoagulant treatment with warfarin (**A**), rivaroxaban (**B**) and LMWH (**C**).

Two studies reported the relationship between warfarin and thrombosis recurrence, and heterogeneity test results showed no significant heterogeneity among studies (*I*^2 ^= 0.000%, *P* > 0.05). (Figure 3A). Meta-analysis was performed using a fixed effect model. The results showed that the pooled proportion of thrombosis recurrence in patients with catheter thrombosis who used warfarin was 0.000 (95% CI: 0.000–0.016) (Figure 3A).

#### 3.3.2. Rivaroxaban

Four studies reported the association between rivaroxaban and VTE recurrence, and heterogeneity test results showed no significant heterogeneity among studies (*I*^2 ^= 0.000%, *P* > 0.05). Meta-analysis was performed using a fixed effect model. The results showed that the pooled proportion of thrombosis recurrence in patients with catheter thrombosis who received rivaroxaban was 0.018 (95% CI: 0.004-0.038) (Figure 3B).

#### 3.3.3. LMWH

Five studies reported the association between LMWH and thrombosis recurrence. The results showed that the pooled proportion of thrombosis recurrence in patients with catheter thrombosis using LMWH was 0.013 (95% CI: 0.001–0.034) (Figure 3). Since the results of the heterogeneity test showed no significant heterogeneity between studies (*I*^2 ^= 19.327%, *p* = 0.292), a fixed effects model was used for the meta-analysis (Figure 3C).

### 3.4. Catheter dysfunction

#### 3.4.1. Warfarin

One study reported an association between warfarin and catheter dysfunction, and heterogeneity testing was not applicable. In this study (12), 42 patients (57%) still had functional central venous catheters in place at 3 months. Catheters were removed before the 3-month end point in 32 patients (43%), but none were removed because of recurrent DVT or line obstruction resistant to tPA infusion. The majority of patients were removed because treatment needed to end.

#### 3.4.2. Rivaroxaban

**Figure 4:**
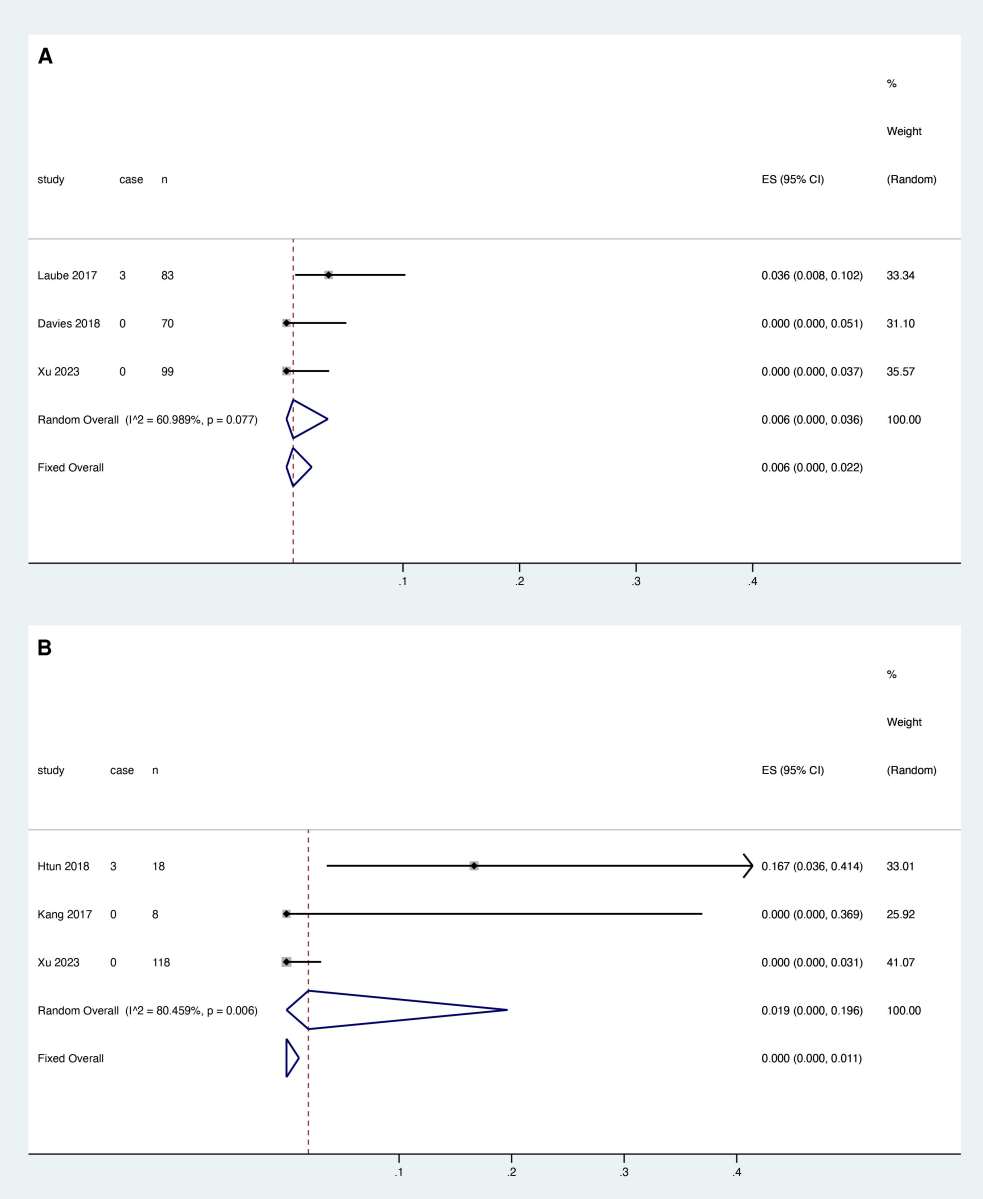
Catheter dysfunction after rivaroxaban (**A**) and LMWH (**B**) treatment.

Three studies reported the association between rivaroxaban and catheter dysfunction, and heterogeneity test results showed that there was heterogeneity among studies (*I*^2 ^= 60.989%, *P* = 0.077). Meta-analysis was performed using a random effect model. The results showed that the pooled proportion of catheter dysfunction was 0.006 with rivaroxaban (95% CI: 0.000–0.036) (Figure 4A).

#### 3.4.3. LMWH

Three studies reported the relationship between LMWH and catheter dysfunction, and the heterogeneity test showed that there was heterogeneity among studies (*I*^2 ^= 80.459%, *P* = 0.006). The random effect model was used for meta-analysis. The results showed that the pooled proportion of catheter dysfunction with LMWH was 0.019 (95% CI: 0.000–0.196) (Figure 4B).

### 3.5. Major bleeding

#### 3.5.1. Warfarin

**Figure 5:**
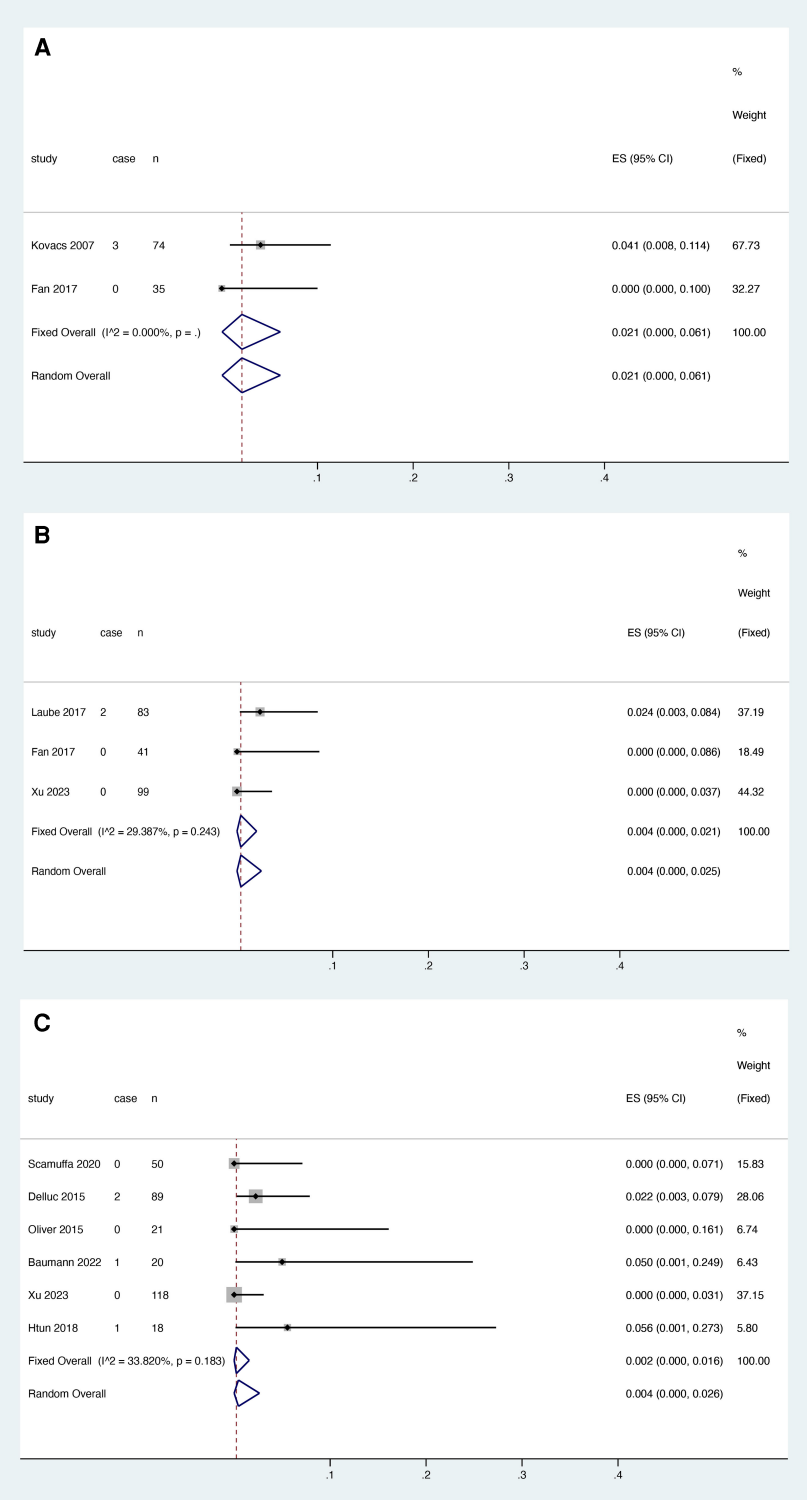
Major bleeding after anticoagulant treatment with warfarin (**A**), rivaroxaban (**B**) and LMWH (**C**).

Two studies reported the relationship between warfarin and major bleeding, and the heterogeneity test results showed that there was no significant heterogeneity among the studies (*I*^2 ^= 0.000%, *P* > 0.05). The fixed effect model was used for meta-analysis. The results showed that the pooled proportion of major bleeding using LMWH was 0.021 (95% CI: 0.000–0.061) (Figure 5A).

#### 3.5.2. Rivaroxaban

Four studies reported the relationship between rivaroxaban and major bleeding, excluding the study by Davies et al. This may be related to the bias introduced by the inclusion of only breast cancer patients in this study (17) and the fact that antitumor drugs taken by breast cancer patients have been reported to potentially increase the risk of exposure to DOACs (23). Heterogeneity decreased from 78.876% to 29.287% (*P* = 0.243), and three studies were finally included. Meta-analysis was performed using a fixed effect model. The results showed that the pooled proportion of major bleeding with rivaroxaban was 0.004 (95% CI: 0.000–0.021) (Figure 5B).

#### 3.5.3. LMWH

Six studies reported the relationship between LMWH and major bleeding, and heterogeneity test results showed no significant heterogeneity among studies (*I*^2 ^= 33.820%, *P* = 0.183). A fixed effect model was used for the meta-analysis. The results showed that the pooled proportion of major bleeding using LMWH was 0.002 (95% CI: 0.000–0.016) (Figure 5C).

### 3.6. Death

#### 3.6.1. Warfarin

**Figure 6:**
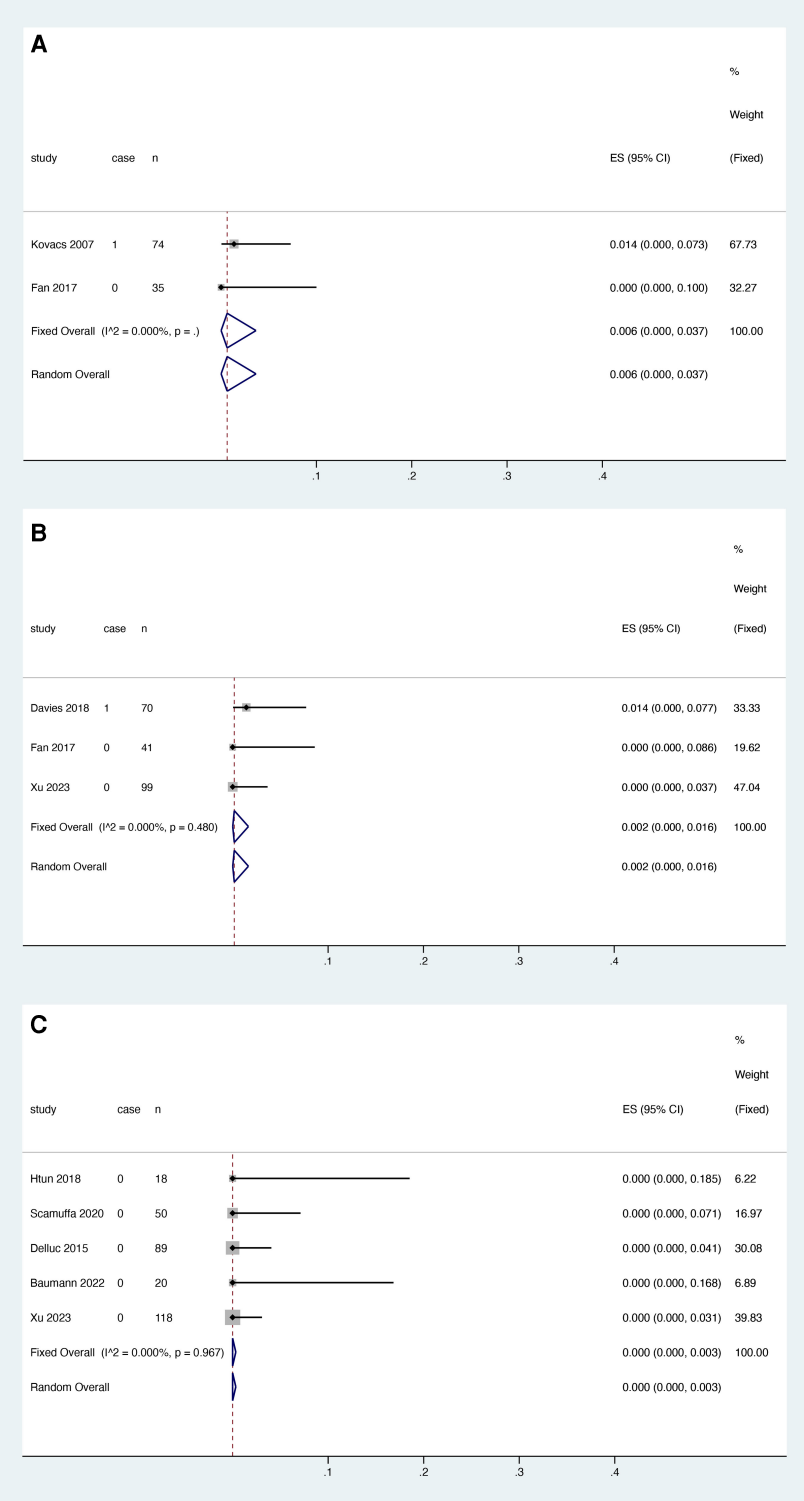
Death after anticoagulant treatment with warfarin (**A**), rivaroxaban (**B**) and LMWH (**C**).

Two studies reported the relationship between warfarin and death, and heterogeneity test results showed no significant heterogeneity among studies (*I*^2 ^= 0.000%, *P* > 0.05). Meta-analysis was performed using a fixed effect model. The results showed that the pooled proportion of deaths with warfarin use was 0.006 (95% CI: 0.000–0.037) (Figure 6A).

#### 3.6.2. Rivaroxaban

Four studies reported the association between rivaroxaban and death, and heterogeneity decreased from 71.626% to 0.000% (*P* > 0.05) after deleting the study by Laube et al. In the study by Laube et al., the cause of death of the patients who died was not stated, we could not judge whether it was retained or not, and the study was removed due to the high degree of heterogeneity created. Meta-analysis was performed using a fixed effect model. The results showed that the pooled proportion of deaths with rivaroxaban was 0.002 (95% CI: 0.000–0.016) (Figure 6B).

#### 3.6.3. LMWH

Six studies reported the relationship between LMWH and death, and heterogeneity decreased from 80.724% to 0.000% (*P* > 0.05) after deleting Oliver et al. In the study by Oliver et al., although the mortality rate of patients in the LMWH-using group was higher than that in the other studies included in this review, it was lower than the mortality rate in the nonanticoagulation group in the text (71%) (19). The survival prognosis of Oliver's findings appeared to be worse than that of Htun's findings without saying what caused the patient's cause of death. Therefore, this analysis excluded the study of Oliver et al. and finally included five studies, which were analyzed by meta-analysis using a fixed-effects model. The results showed that the pooled proportion of deaths using LMWH was 0.000 (95% CI: 0.000–0.003) (Figure 6C).

### 3.7. Subgroup

#### 3.7.1. VTE recurrence

**Figure 7:**
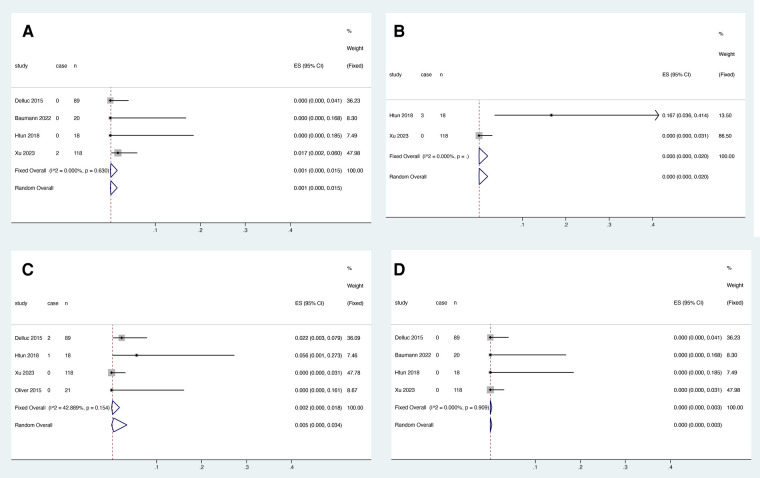
VTE recurrence (**A**), catheter dysfunction (**B**), major bleeding (**C**) and death (**D**) within three months of LMWH treatment.

Four studies reported the association between LMWH and VTE recurrence within the first three months, and heterogeneity test results showed no significant heterogeneity among studies (*I*^2 ^= 0.000%, *P* = 0.630). Meta-analysis was performed using a fixed effect model. The results showed that the pooled proportion of VTE recurrence with rivaroxaban was 0.001 (95% CI: 0.000–0.015) (Figure 7A).

#### 3.7.2. Catheter dysfunction

Two studies reported an association between LMWH and catheter dysfunction, and heterogeneity test results showed no significant heterogeneity among studies (*I*^2^ = 0.000%, *P* > 0.05). Meta-analysis was performed using a fixed effect model. The results showed that the pooled proportion of catheter dysfunction with rivaroxaban was 0.000 (95% CI: 0.000–0.020) (Figure 7B).

#### 3.7.3. Major bleeding

Four studies reported the association between LMWH and major bleeding within the first three months, and heterogeneity test results showed no significant heterogeneity among studies (*I*^2 ^= 42.889%, *P* = 0.154). Meta-analysis was performed using a fixed effect model. The results showed that the pooled proportion of major bleeding with rivaroxaban was 0.002 (95% CI: 0.000–0.018) (Figure 7C).

#### 3.7.4. Death

Three studies reported the association between rivaroxaban and death within the first three months, and heterogeneity test results showed no significant heterogeneity among studies (*I*^2 ^= 0.000%, *P* > 0.05). Meta-analysis was performed using a fixed effect model. The results showed that the pooled proportion of death with rivaroxaban was 0.000 (95% CI: 0.000–0.003) (Figure 7D).

## 4. Discussion

Cancer patients are at high risk of developing venous thrombosis due to a tendency toward hypercoagulation, and the insertion of foreign objects into veins which further increases the risk (24). The goals of managing catheter-related UEVTE include relieving symptoms, preventing embolism, and maintaining continuous venous access (25). Our review included 770 patients from 11 studies in who were meta-analyzed. This review focuses on the effectiveness of outcomes including VTE recurrence and safety outcomes including catheter dysfunction, major bleeding, and death. The results of the analyses are presented as pooled proportions of anticoagulant effectiveness and safety events. The analysis revealed that in terms of effectiveness, warfarin had the lowest incidence of VTE recurrence at 0.000, compared to 0.018 for rivaroxaban and 0.013 for LMWH. Regarding safety, the pooled proportions of risk of major bleeding and death for LMWH were 0.002 and 0.000, respectively, which were smaller than those for warfarin and rivaroxaban, but the risk of catheter dysfunction was the highest at 0.019. For rivaroxaban vs. warfarin, the pooled proportions of major hemorrhage and death events were lower for rivaroxaban than for warfarin (0.004 vs. 0.021 and 0.002 vs. 0.006, respectively), but the pooled proportions of catheter dysfunction events were higher for rivaroxaban than for warfarin (0.006 vs. 0.000). Since the follow-up period for both rivaroxaban and warfarin was within three months, whereas the follow-up period for LMWH was up to 2,495 days, to avoid bias associated with the duration of follow-up, we extracted the available outcome data within three months of follow-up in the LMWH group and used them for subgroup analyses. Compared with the direct analysis of LMWH, the pooled proportions of catheter dysfunction events, major hemorrhage, and death did not change in the subgroup analyses, but the risk of recurrent VTE events was lower (from 0.013 to 0.001), which makes it appear that LMWH has a greater advantage in terms of efficacy.

CRTs are a common complication; they are usually nonprogressive and may resolve with CVC removal (26). However, central venous catheters play a key role in the management of hospitalized cancer patients (27). Guidelines mention that in most patients with UEDVT associated with central venous catheters, catheter removal should not occur if the catheter is functional and is continuously needed (28). A recent retrospective study found that catheter removal prior to anticoagulation was associated with an increased risk of VTE recurrence. Therefore, in cases where immediate removal is not needed, it may be beneficial to delay catheter removal until the patient can be properly anticoagulated (29). Some guidelines have suggested that anticoagulation should be administered for at least 3 months and for the duration of the central venous catheter placement; in this case, LMWHs are recommended (30–32). The pooled rate of catheter dysfunction was 0.019 for LMWH and 0.006 for rivaroxaban. The higher risk of catheter dysfunction for LMWH in this review appears to be contrary to guideline recommendations. However, the results are interpretable. The LMWH group included three studies for the analysis of outcomes of catheter-related disorders. The studies by Kang et al. (21) and Xu et al. (22) had no patients with catheter dysfunction, whereas the study by Htun et al. (33) included 18 patients with acute leukemia, of whom three had their catheters removed due to wound infections and culture-negative sepsis, respectively. It is well known that patients with hematological malignancies are at higher risk of infection than patients with other tumors, given their immunosuppression and the widespread use of blood transfusions in this population (34). Consequently, the high risk of catheter dysfunction in the LMWH group may be attributable to the tumor type rather than the coagulant.

Although LMWH is the gold standard of care for cancer-associated thrombosis (CAT), there are limitations on its use due to cost; therefore, warfarin is commonly prescribed (35). The NCCP guidelines offer warfarin as an alternative to LMWH for the treatment of VTE, which avoids the psychological burden associated with daily injections compared to LMWH. A recent meta-analysis compared the efficacy and safety of warfarin and LMWH in patients with cancer-related thrombosis, with data from six collected studies demonstrating the effectiveness of LMWH in reducing VTE over warfarin [risk ratio (RR): 0.67; 95% CI: 0.47–0.95; *p* = 0.03]. However, LMWH was similar to warfarin in major bleeding (RR: 1.05; 95% CI: 0.62–1.77; *p* = 0.85), minor bleeding events (RR: 0.80; 95% CI: 0.54–1.20; *p* = 0.28) and all-cause mortality (RR: 1.00; 95% CI: 0.88–1.13; *p* = 0.99) (36). The combined effect of major bleeding (0.021) and death (0.006) appeared to be higher for warfarin in this study. There are several reasons that might explain this. The use of VKAs requires frequent monitoring and dose adjustments to maintain the international normalized ratio (INR) within the therapeutic range (37). Obtaining sufficient time in the therapeutic INR range (TTR) for CAT (cancer-associated venous thromboembolism) patients can be challenging; the TTR was 47.0% in the CATCH study and 46% in the CLOT study (38). In addition, warfarin is more likely to have drug‒drug interactions (DDI) with anticancer drugs than LMWH because the metabolism of warfarin interacts with a variety of CYP enzymes (2C9, predominantly), whereas the metabolism of enoxaparin is not related to the CYP enzyme system drug (39). These features induce anticoagulation with warfarin in CRT patients which is a requirement for more comprehensive coagulation index testing and DDI risk monitoring, which may explain the high pooled rates of major bleeding and death in the warfarin group in this study. Due to the limited number of studies in the warfarin group, the meta-analysis results should be interpreted with caution.

Rivaroxaban is a new oral anticoagulant with advantages over warfarin, including no laboratory monitoring, reduced drug‒drug interactions, and reduced food-drug interactions. In a retrospective study based on Surveillance, Epidemiology, and End Results (SEER)-Medicare linked data, it was found that rivaroxaban was associated with a reduced risk of recurrent VTE vs. LMWH without a significant impact on the composite outcome, major bleeding, or all-cause mortality (40). A meta-analysis comparing the efficacy and risk of DOACs with LMWH in patients with cancer-related VTE found that cancer patients receiving DOACs had significantly fewer recurrences of VTEs and no increased risk of major bleeding (41). In this review, rivaroxaban did not show significant benefits in terms of efficacy and safety, specifically, the prevention of VTE recurrence and cause of catheter dysfunction, with suboptimal results in the rivaroxaban group. Although rivaroxaban is inferior to LMWH in patients with CAT in the meta-analysis by Dong et al., a catheter complicates the process of anticoagulation in patients with cancer, and therefore, the use of DOACs in cancer patients with CRT needs to be supported with additional evidence.

It is important to consider the limitations of this study. First, this study is a systematic review and meta-analysis based on observational cohort studies. Nine of the 11 included studies were single-armed and lacked direct comparisons between each, resulting in a lower quality of evidence. Further explanation of the findings of this study is needed. Moreover, there may be significant differences in baseline characteristics, including the type of cancer and catheter and whether the patient had the catheter removed. The limited number of included studies made it difficult to stratify studies based on the aforementioned confounding factors.

In conclusion, future long-term randomized controlled trials are recommended to further clarify the relationship between the safety and efficacy of different anticoagulants for catheter-related thrombosis.

## 5. Conclusions

This meta-analysis demonstrated the efficacy and safety advantages of LMWH at three months after CRT. In cancer patients, LMWH remains a frequent option for the treatment of CRT. In summary, anticoagulation in cancer patients with CRT remains a considerable problem, and more clinical studies are needed to improve the quality of evidence for anticoagulation recommendations in CRT cancer patients.

## Data Availability

The original contributions presented in the study are included in the article/Supplementary Material, further inquiries can be directed to the corresponding author.
