# Supplementary material for: Outcome of antithrombotic therapy in cancer patients with catheter-related thrombosis: a systematic review
Source: Front Cardiovasc Med. 2023 Dec 12;10:1290822. doi: 10.3389/fcvm.2023.1290822 (PMC10756912; doi:10.3389/fcvm.2023.1290822)
Supplement: Supplementary file 1 [file Datasheet1.docx]

**Supplementary material**

**
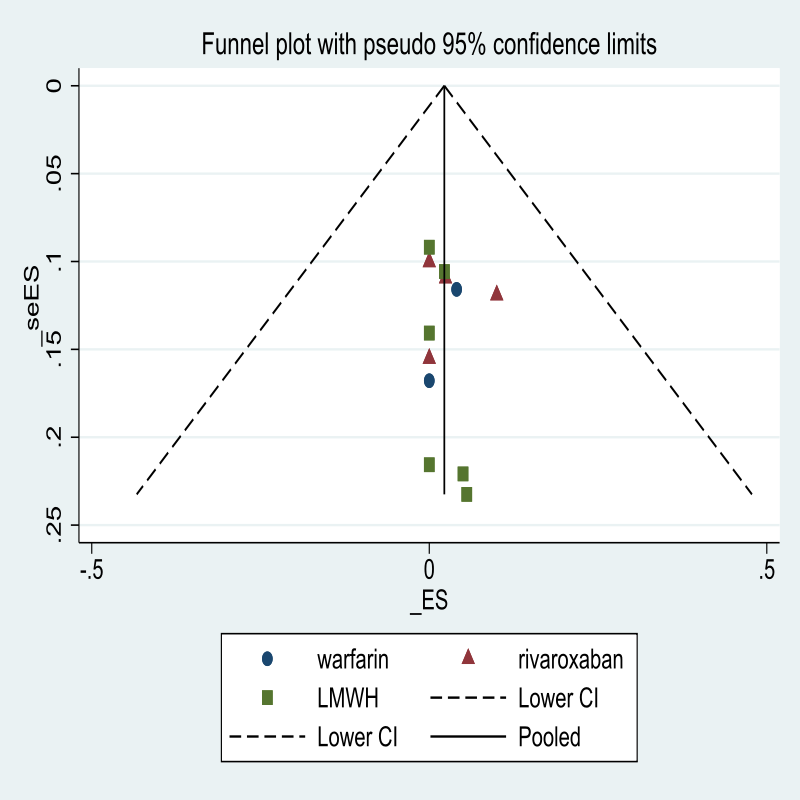
**

Supplementary FIG 1：Begg's funnel plot for major bleeding


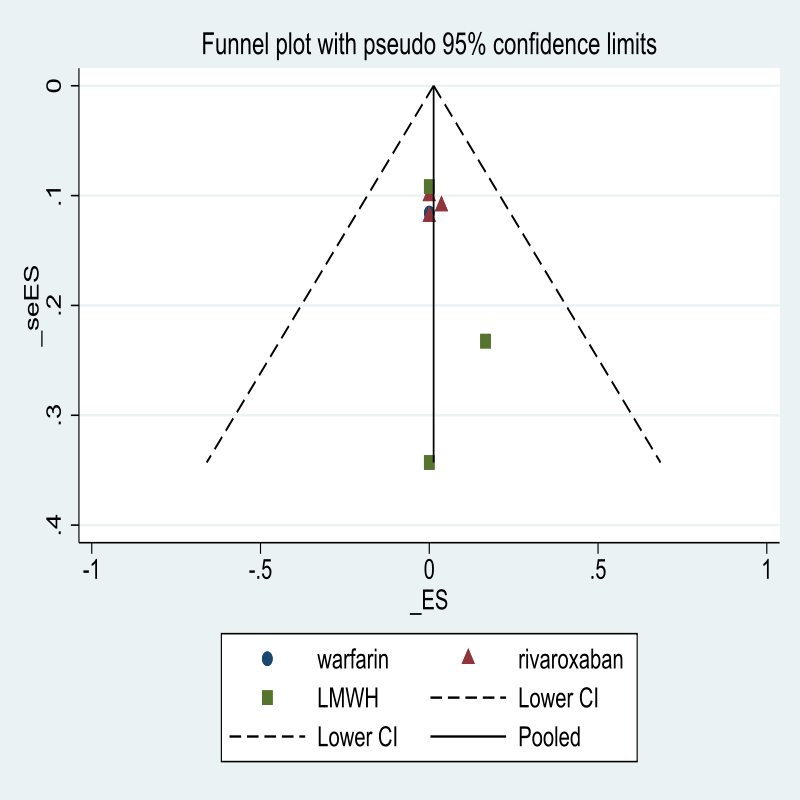


Supplementary FIG 2：Begg's funnel plot for catheter dysfunction


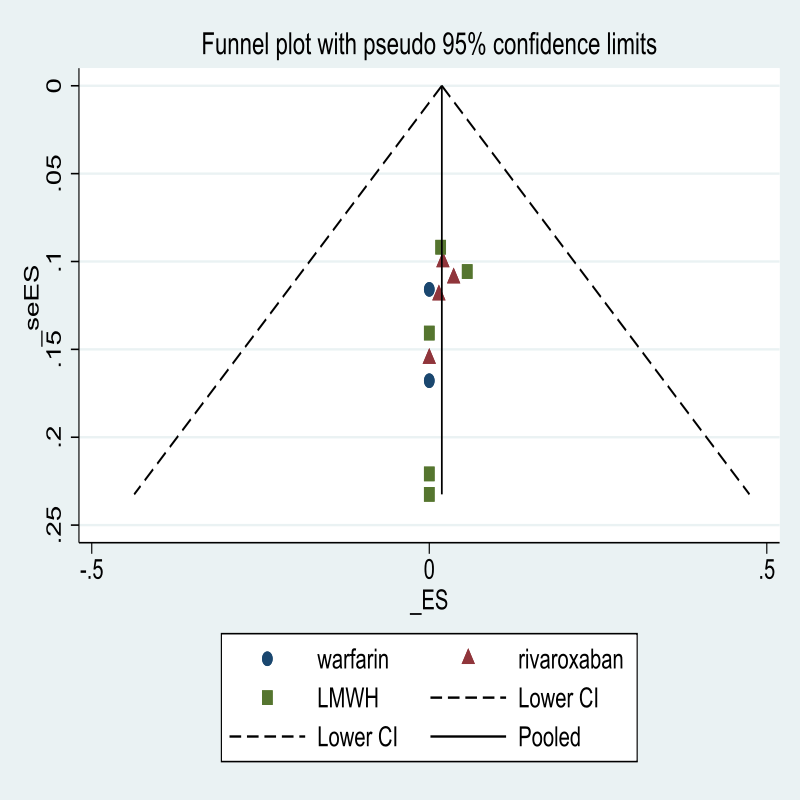


Supplementary FIG 3：Begg's funnel plot for VTE recurrence


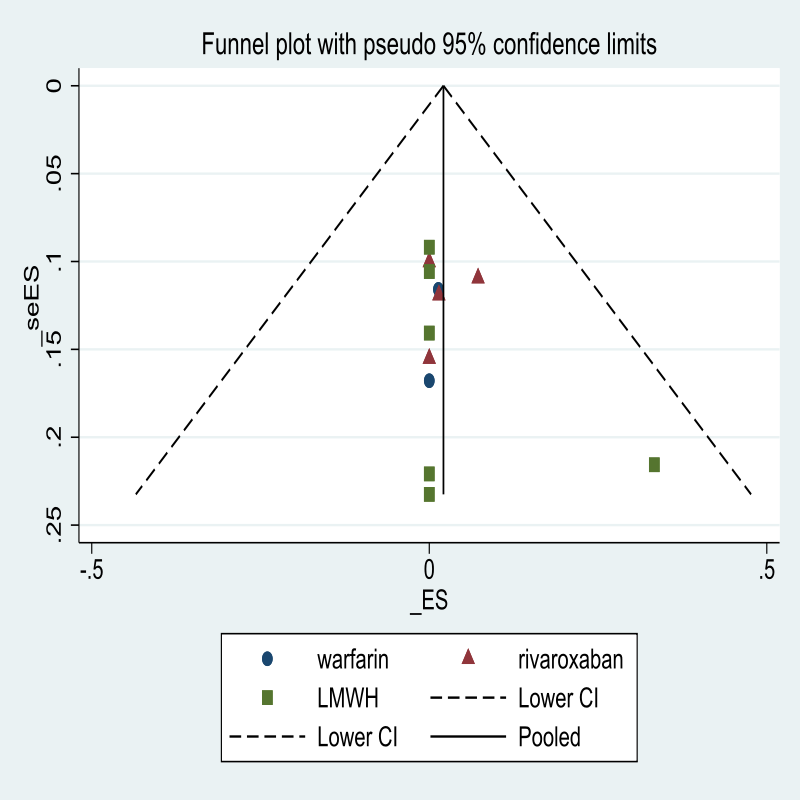


Supplementary FIG 4：Begg's funnel plot for death
